# Systematic identification of key functional modules and genes in esophageal cancer

**DOI:** 10.1186/s12935-021-01826-x

**Published:** 2021-02-25

**Authors:** Rui Wu, Hao Zhuang, Yu-Kun Mei, Jin-Yu Sun, Tao Dong, Li-Li Zhao, Zhi-Ning Fan, Li Liu

**Affiliations:** 1 Department of Digestive Endoscopy, The First Affiliated Hospital with Nanjing Medical University, 300 Guangzhou Road, Nanjing, 210029 Jiangsu China; 2 Nanjing Medical University, 101 Longmian Avenue, Nanjing, Jiangsu China; 3 Department of Cardiology, The First Affiliated Hospital with Nanjing Medical University, 300 Guangzhou Road, Nanjing, 210029 Jiangsu China

**Keywords:** Esophageal cancer, Integrated transcriptomic analysis, Weighted gene co-expression network analysis, Bioinformatics, CCNB1

## Abstract

**Background:**

Esophageal cancer is associated with high incidence and mortality worldwide. Differential expression genes (DEGs) and weighted gene co-expression network analysis (WGCNA) are important methods to screen the core genes as bioinformatics methods.

**Methods:**

The DEGs and WGCNA were combined to screen the hub genes, and pathway enrichment analyses were performed on the hub module in the WGCNA. The *CCNB1* was identified as the hub gene based on the intersection between DEGs and the greenyellow module in WGCNA. Expression levels and prognostic values of *CCNB1* were verified in UALCAN, GEPIA2, HCMDB, Kaplan–Meier plotter, and TIMER databases.

**Results:**

We identified 1,044 DEGs from dataset GSE20347, 1,904 from GSE29001, and 2,722 from GSE111044, and 32 modules were revealed by WGCNA. The greenyellow module was identified as the hub module in the WGCNA. *CCNB1* gene was identified as the hub gene, which was upregulated in tumour tissues. Moreover, esophageal cancer patients with higher expression of *CCNB1* showed a worse prognosis. However, *CCNB1* ‘might not play an important role in immune cell infiltration.

**Conclusions:**

Based on DEGs and key modules related to esophageal cancer, *CCNB1* was identified as the hub gene, which offered novel insights into the development and treatment of esophageal cancer.

## Introduction

Esophageal cancer (ESCA) is the seventh most common cancer and the sixth leading cause of cancer-related death worldwide [1, 2]. In 2018, there were 572,034 new victims and 508,585 deaths globally, and almost half of the cases occur in China [1, 3]. The 5-year survival rate of early-stage cancer may reach 85%, while it drops to less than 15% when the cancer progress to advanced stage [4, 5]. Despite the rapid development of multimodal treatments in recent years, esophagectomy remained the standard curative method for advanced ESCA, and patients still experience poor life quality and face risk of long-term recurrence [6–8].

Currently, clinical screening of early ESCA mainly depends on endoscopic observation combined with biopsy-based histopathological diagnosis, however, it can find only 19% of ESCA at early stages [9]. The reliability of such approaches encounter limitations due to variation in endoscopic sampling site and observer’s experience, and may lead to unnecessary biopsies, costs, and high false-positive rates. Even the molecular and cellular changes may appear indolent in endoscopic histology to avoid surveillance [10, 11]. Thus, it is urgent to explore novel approaches or biomarkers to ameliorate the early detection, treatment guidance, and prognosis prediction for patients. Currently, several researchers have reported their exploration of biomarkers for esophageal cancer and their effects [12–14]. Defects of a mitotic checkpoint may bring about mistakes in the chromosome segregation, and the higher level of *Cyclin B1* (*CCNB1*) is a marker of poor prognosis in many cancer types [15, 16]. However, there are few reports about *CCNB1* as a biomarker of ESCA.

Recently, many researchers focus on microarray analysis of gene expression datasets. They succeeded in revealing characteristic genes with the involved key pathways as new cancer biomarkers, with the use of differentially expressed genes(DEGs)and weighted gene co-expression network analysis (WGCNA) [17, 18]. WGCNA tends to identify a co-regulated transcriptional profile of functional gene assemblies, thus enabling a precise network of hub genes and clinical traits [19]. Our study may help understanding the potential molecular mechanism of ESCA initiation and progression and provide a novel prospect for the clinical diagnosis and treatment in ESCA.

## Methods

### Data acquisition and preprocessing

The expression profile of GSE20347, GSE29001, and GSE111044 was achieved based on gene expression omnibus (GEO, https://www.ncbi.nlm.nih.gov/geo/), which is a public database containing comprehensive data of gene profiling and sequencing. GSE20347 included 17 pair-wise ESCA tissues and normal adjacent tissues. GSE29001 included matched normal basal epithelial cells, normal differentiated squamous epithelium, and ESCA from 12 patients. GSE111044 included 3 ESCA tissues and corresponding 3 normal tissues from 3 patients. The experiment GSE20347 and GSE29001 were conducted on platform GPL571 (Affymetrix Human Genome U133A 2.0 Array), and GSE111044 was on platform GPL570 (Affymetrix Human Genome U133 Plus 2.0 Array). After eliminating redundant data (e.g., null value, time), all the gene symbols were matched with probes and were subsequently screened with the ‘limma’ package of R software 3.4.1 to perform background correction, quartile normalization, and quantiles summarization.

### Identification of DEGs between ESCA and normal tissues

In this study, we utilized the ‘limma’ package in bioconductor (http://www.bioconductor.org/) to uncover DEGs between normal and ESCA tissues. Adjusted *P* < 0.05 and |log2 fold change| > 1 was set as the criteria for selecting significant DEGs according to the normalized gene expression levels.

### Establishment of WGCNA on ESCA

WGCNA is regard to a methodology to reconstruct a free-scale gene co-expression network and concurrently identify modules consisted of highly correlated genes to appraise connectivity between external clinical traits and the module, in which eigengene is used for summarizing relationship among internal gene membership. In the study, we applied the one-step network construction and module detection function of WGCNA package in R to handle the analysis of microarray dataset GSE70409, which contains 17 cancerous tissues and 17 normal tissues. First, a weighted adjacency matrix was calculated to represent the connecting strength over each pair of gene with outliers removed and ensure a co-expression network. The soft thresholding power was set as 7 to obtain a scale‑free topology network. Then, a hierarchical clustering dendrogram was established constituted of abundant branches, and each branch was assigned with a colour to reveal a module. Finally, the modules were used to the association with clinical traits by using module-trait associations according to module membership (MM) and gene significance (GS). Moreover, topological overlap matrix method was utilized for verifying correlation character of eigengenes in different modules.

### Module preservation evaluation and principal component analysis (PCA) analysis

Zsummary is usually used to evaluate the preservation of modules. It takes into consideration several statistics, such as the density and connectivity patterns of module nodes, as well as the overlap among module membership. However, a huge difference in module size is easy to induce deviation in Zsummary value. In our study, the green module had far more genes than the greenyellow module, and consequently, we adopted medianRank, since it eliminates the impact of module size. The module with a lower medianRank has more preservation value than that with a higher medianRank. The finally preserved greenyellow module was processed with PCA to examined the ability of gene in the module to discriminate tumor tissues from normal tissues. PCA was conducted through ‘gmodels’ and ‘scatterplot3d’ in R software.

### Pathway enrichment analyses of genes in the hub module

Gene ontology (GO) is a common method for gene analysis [20]. In the study, we used GO analysis to classify the genes in greenyellow module into three categories based on their bio-function, including biological process (BP), cellular component (CC), and molecular function (MF). Meanwhile, Kyoto encyclopedia of genes and genomes (KEGG) pathway enrichment analysis was also performed for exploration of their biological characteristics. Both the GO and KEGG pathway analyses were conducted using the ‘clusterProfiler’ package in R, with adjusted *P*-value of the analysis calculated by the Benjamini and Hochberg false discovery rate algorithm. Furthermore, a circular chordal graph of the data was generated utilizing gene network analysis via the default euclidean distance and average linkage.

### Extract of hub genes from DEGs and the hub module in WGCNA

39 candidate genes were screened out from the intersection of venn diagram between 3 set of DEGs and the greenyellow module in WGCNA (Fig. 5). *CCNB1* was chosen for the next research for the most significant *P* value. For further study, the protein–protein interaction (PPI) network was constructed among 39 candidate genes (string, https://string-db.org/), and Cytoscape software was applied to visualize the PPI network.

### Verification of gene aberrant expression in UALCAN, GEPIA2, and HCMDB

UALCAN (http://ualcan.path.uab.edu/index.html) is a comprehensive web resource for analyzing cancer OMICS data with the use of TCGA and MET500 databases. In our study, *CCNB1* expression data was obtained through ‘Expression Analysis’ module for the contrast of promoter methylation and gene overall expression level. Further stratified analysis based on gender, age, cancer stages, and histology was also conducted. Additionally, GEPIA2 (http://gepia2.cancer-pku.cn/) and Human Cancer Metastasis Database (HCMDB, http://hcmdb.i-sanger.com/index) analysis was performed to explore the differential expression of *CCNB1* between normal tissues and ESCA tissues. GEPIA2 is based on CGA and the GTEx databases with a total of 84 cancer subtypes analysis. HCMDB is designed to examine gene expression of primary and metastasis tumour from 124 previously published transcriptome datasets from TCGA and GEO databases.

### Survival analysis with Kaplan–Meier (KM) plotter database

KM plotter (http://kmplot.com/) was used to plot the overall survival status and estimate prognostic valve of *CCNB1*, which is an interactive website containing 54 k genes impact data on survival of 21 cancer types. According to median gene expression level, all patients’ survival data was divided into two group: high expression group and low expression group. The *P* value was set < 0.05 to ensure statistically significance.

### Tumor infiltration analysis of TIMER database

TIMER database provides a systematical analysis of infiltrating abundances in 6 types of immune cells (B cells, CD4+ T cells, CD8+ T cells, neutrophils, macrophages, and dendritic cells) and the infiltration relevant clinical outcome. Hence, we adopted this method to research the tumor infiltration as well as the survival data on *CCNB1*.

## Results

### DEGs screening from normal and cancerous tissues

**Fig. 1:**
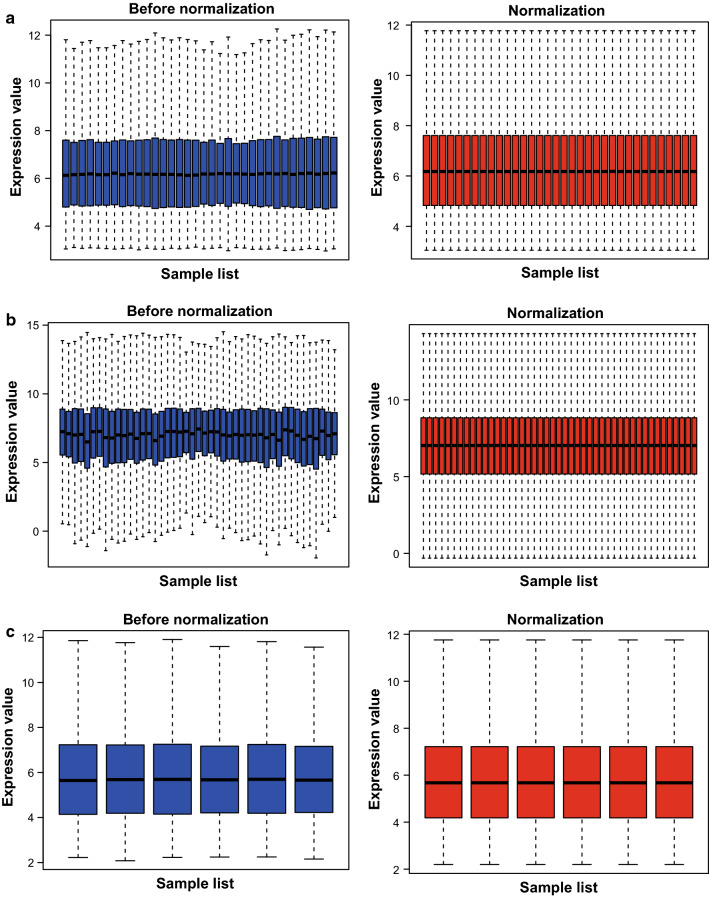
Identification of DEGs. Box plot of genes expression data before and after normalization in GSE20347 (**a**), GSE29001 (**b**), and GSE111044 (**c**)

**Fig. 2:**
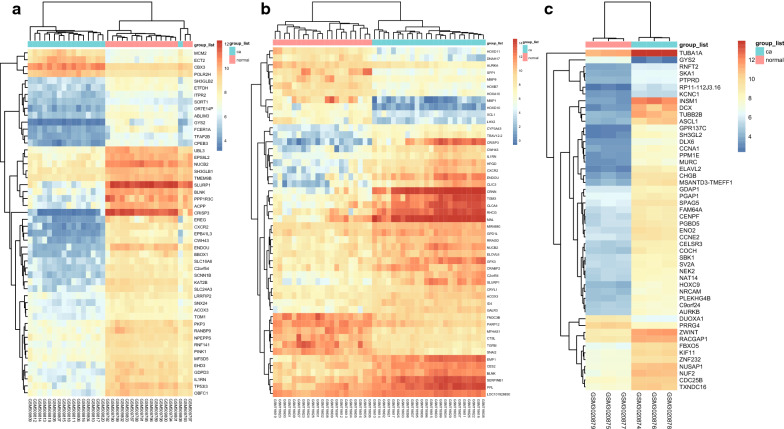
Heatmap of DEGs. **a** Heatmap of DEGs in GSE20347. **b** Heatmap of DEGs in GSE29001. **c** Heatmap of DEGs in GSE111044

After preprocessing and normalization (Fig. 1a–c), 1044 DEGs from dataset GSE20347, 1904 from GSE29001, and 2722 from GSE111044 were respectively identified by comparing their expression in cancerous esophageal tissues with normal tissues. As is shown in the volcano plot, there were 571 upregulated and 473 downregulated genes observed in the GSE20347 dataset (Additional file 1: Fig. S1A). Whereas for the dataset GSE29001 and GSE111044, the amount was 894 upregulated genes, 1010 downregulated genes, and 1370 upregulated genes, 1352 downregulated genes, separately (Additional file 1: Fig. S1B, C). The heatmap of upregulated and downregulated genes were shown in Fig. 2a, b.

### WGCNA for genes in GSE70409 dataset

**Fig. 3:**
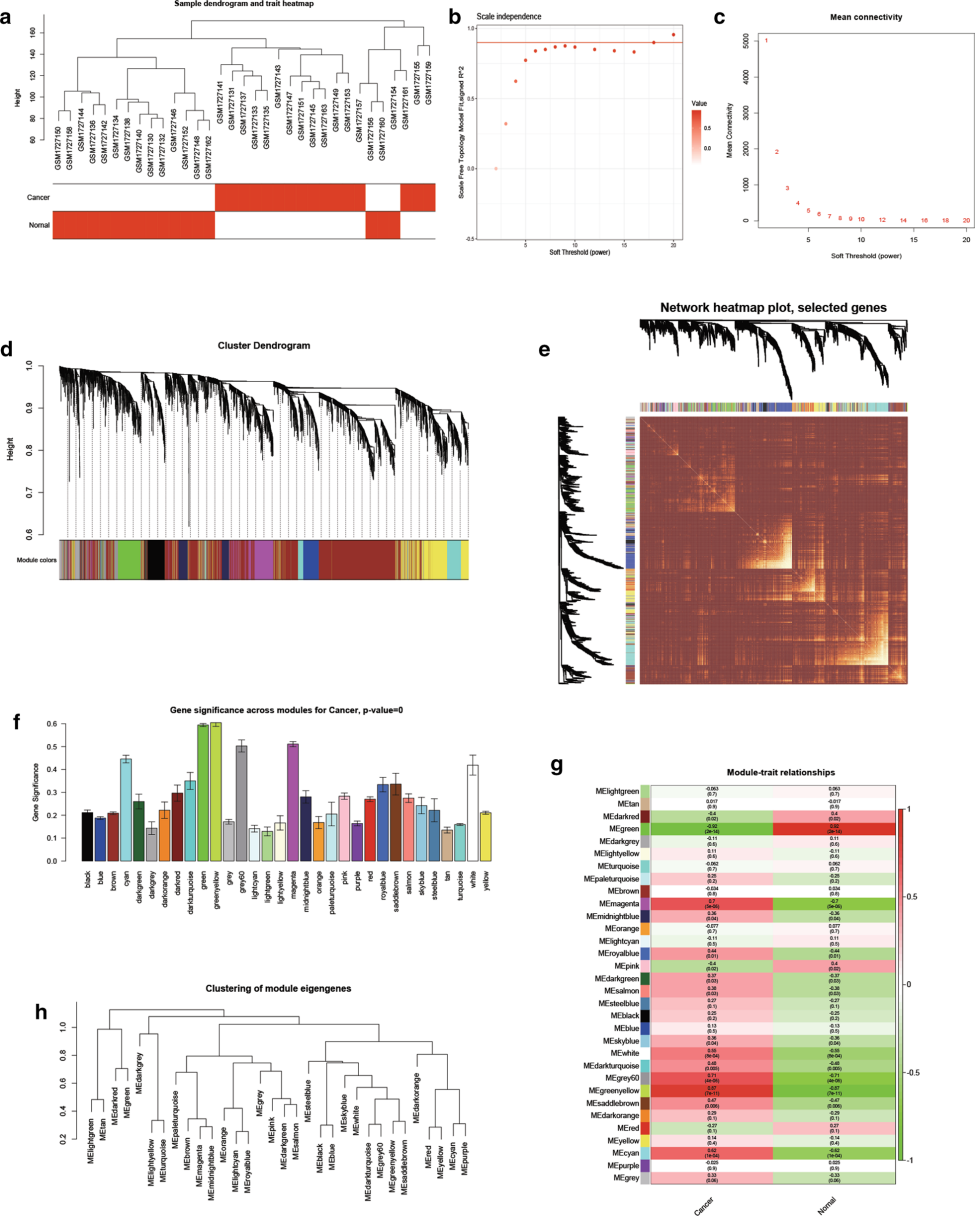
WGCNA analysis. **a** Clustering dendrogram of samples with trait heatmap. **b** Analysis of the scale-free fit index for various soft-thresholding powers (β). **c** Analysis of the mean connectivity for various soft-thresholding powers. **d** Cluster dendrogram of genes based on dissimilarity of topological overlap. Each module is assigned a colour, with each branch representing a gene. **e** Network heatmap based on the selected genes. **f** Gene significance distribution of modules. **g** Module-trait heatmap of the correlation between module eigengenes and clinical traits of ESCA. **h** Cluster dendrogram of eigengenes in modules

We utilized WGCNA package in R software to build a weighted co-expression network. The samples of GSE70409 were clustered to filter outlier for subsequent analysis, and one discrete sample (GSM1727139) was noticed and removed out. (Fig. 3a). In our study, the power of β = 7 (scale-free R2 = 0.95) was chosen as the soft-thresholding parameter to ensure a scale-free network (Fig. 3b, c). A total of 32 modules were identified from 19,827 genes, and each module was assigned a colour in hierarchical clustering dendrogram (Fig. 3d). The heatmap was plotted to indicate the similarity of co-expression genes at the network topology level (Fig. 3e). The green module (containing 1906 genes) and greenyellow module (containing 469 genes) were found to have the most prominent module significance (Fig. 3f), and they all shown great clinical meaning (green: correlation coefficient = − 0.92, *P* < 0.01; greenyellow: correlation coefficient = 0.87, *P* < 0.01; Fig. 3g). Clustering of module eigengenes illustrated that the green and greenyellow modules were derived from different meta-modules (Fig. 3h**)**. Consequently, the green and greenyellow modules were set as candidate modules for further identification.

### Identification of key module and PCA analysis

**Fig. 4:**
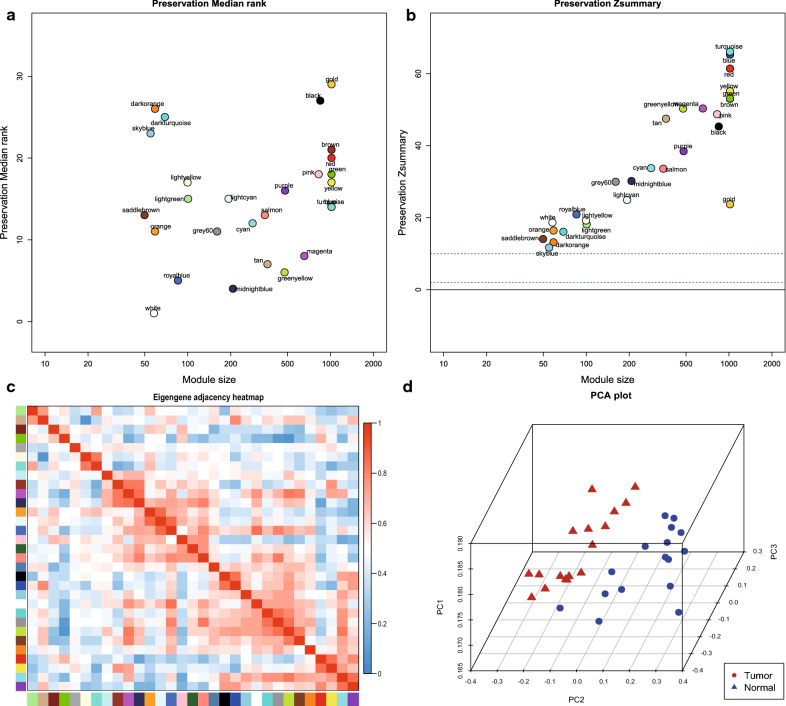
Module preservation evaluation and PCA. **a** MedianRank score of different modules. The lower score, the more preservation value. **b** Zsummary score of different modules. The higher score, the more preservation value. **c** Eigengene adjacency heatmap. **d** The genes in the purple module were shown the degree of overlapping between samples in each of the normal and tumour by using PCA

In view of the large difference of genes amount in green module than in greenyellow module, we adopted medianRank with replace of Zsummary to conduct module preservation, because medianRank is more stable in this situation. The result demonstrated that the greenyellow module had a lower medianRank than green module, so it was selected as the key module (Fig. 4a, b). The greenyellow module presented a bunching relationship with purple module in the eigengene adjacency heatmap (Fig. 4c). In addition, the result of PCA indicated a satisfactory concentration of genes within greenyellow module and the great ability to distinguish tumor from normal tissues (Fig. 4d).

### GO and KEGG analysis of key module and PPI network construction

**Fig. 5:**
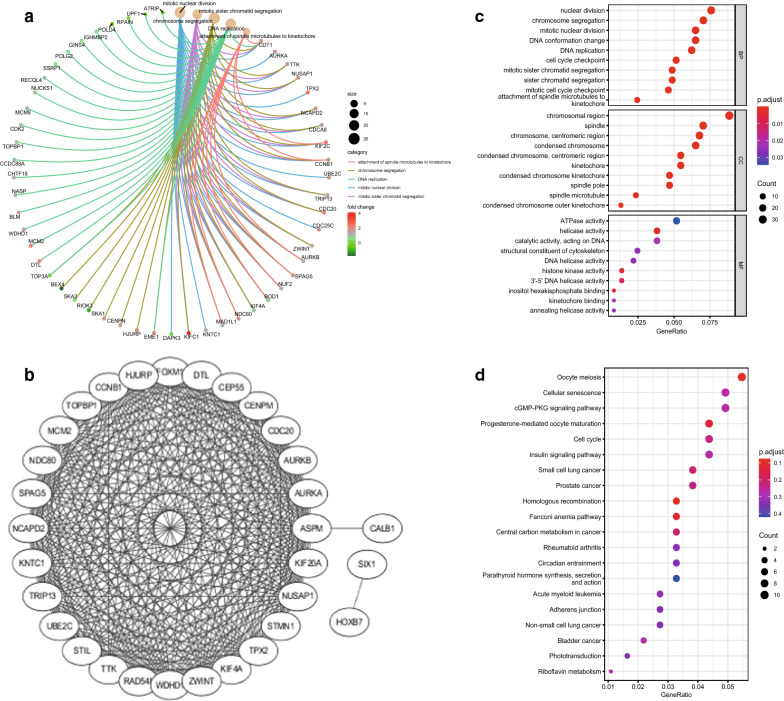
GO and KEGG enrichment analysis and PPI network. **a** Chordal graph visualization of part GO analysis. **b** Protein–protein interaction (PPI) network of 39 joined genes. **c** GO analysis of genes in key module, through 3 dimensions including biological process (BP), cellular component (CC), and molecular function (MF). **d** KEGG enrichment analysis of genes in key module

In order to take a deeper insight into the biological function of these genes in greenyellow module, we used the ‘clusterProfiler’ package in R to perform the GO and KEGG pathway analyses. The noteworthy pathway of GO analysis is visualized through a chordal graph (Fig. 5a). As presented in Fig. 5c, in BP group, genes were primarily involved in chromosome segregation, mitotic nuclear division, DNA replication, and mitotic sister chromatid segregation; in CC group, genes were markedly enriched in chromosomal region, spindle, chromosome centromeric region, and condensed chromosome; in MF group, the significantly enriched pathway were helicase activity, structural constituent of cytoskeleton, histone kinase activity, and 3’-5’ DNA helicase activity. According to KEGG pathway analysis, the most markedly enriched pathways included oocyte meiosis, cellular senescence, progesterone-mediated oocyte maturation, and cell cycle (Fig. 5d).

Furthermore, the venn diagram showed the intersection 39 genes of DEGs and key module (Additional file 2: Fig. S2), and The *CCNB1* was found as hub genes based on the *P*-value. A PPI network was built for those 39 genes (Fig. 5b).

### The expression level, survival analysis, and immune infiltration abundance analysis of CCNB1 in patients with ESCA

**Fig. 6:**
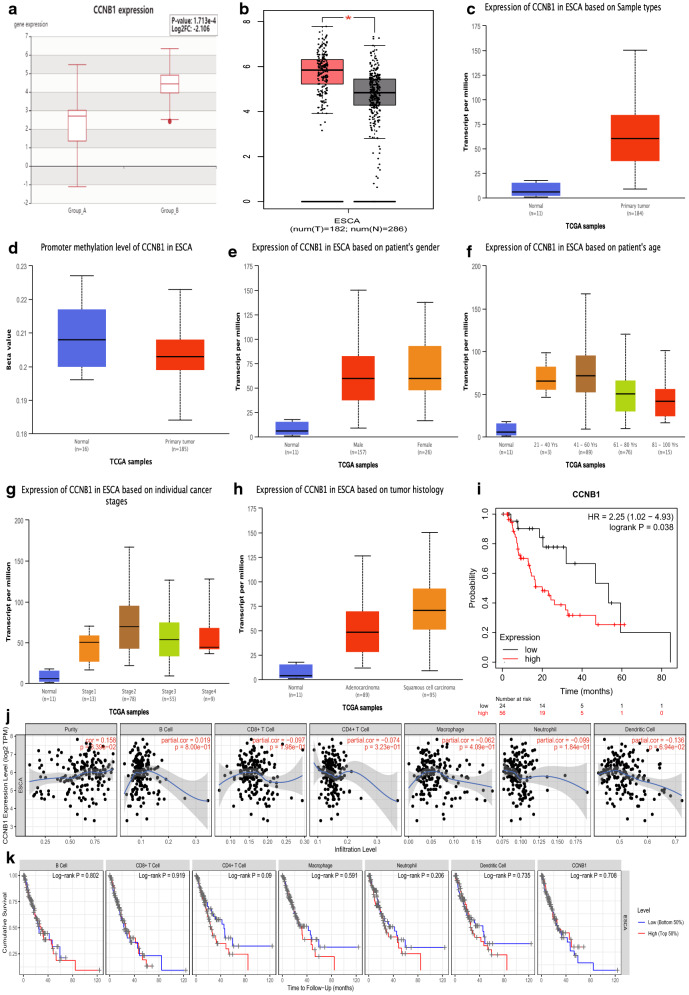
The expression level, survival analysis, and immune infiltration abundance analysis of CCNB1 in patients with ESCA. **a** HCMDB analysis of aberrant expression of *CCNB1* in ESCA patients. **b** GEPIA2 analysis of aberrant expression of *CCNB1* in ESCA patients. **c** UALCAN analysis of aberrant expression of *CCNB1* in ESCA patients. **d** UALCAN analysis indicated downregulated promoter methylation level of *CCNB1* in ESCA patients. **e**–**h** The different expression level of *CCNB1* based on patients’ gender, age, stages, and histology were explored in ESCA patients using UALCAN website. **i** K-M curve revealing the prognosis valve of *CCNB1* in patients with ESCA. **j** Correlation analysis between different expressed *CCNB1* and immune cell infiltration by utilizing TIMER. **k** Prognosis of ESCA patients with different immune infiltration abundance

The results of GEPIA2, HCMDB, and UALCAN all showed a significantly higher expression level of *CCNB1* for patients with ESCA (Fig. 6a–c, P < 0.01). Promoter methylation level was lower in ESCA patients than in normal people for *CCNB1* (Fig. 6d, P < 0.05). To deeply explore the association between clinicopathological parameters with expression level of *CCNB1*, stratified analysis was performed based on patients’ gender, age, cancer stages, and histology. As is shown in Fig. 6E, *CCNB1* got significantly higher expression in both men and women than in healthy people (*P* < 0.01). People over 40 years old had higher *CCNB1* expression (Fig. 6F, P < 0.01), and the top expression of *CCNB1* occurred in 41–60 years. Compared with healthy people, the expression of *CCNB1* was higher in ESCA patients of stage 1–4 (*P* < 0.01), and reached the highest expression in stage 2 (Fig. 6g). With regard to histology (Fig. 6h), the expression of *CCNB1* in adenocarcinoma or squamous cell carcinoma subtypes was remarkably higher than in normal tissues (*P* < 0.01). Notably, squamous cell carcinoma had more *CCNB1* expression than adenocarcinoma, the difference is significant (*P* < 0.01). KM curve demonstrated the better overall survival of ESCA patients in low-risk group than in high-risk group (HR = 2.25, *P* = 0.038) in Fig. 6i. The correlation among gene expression, immune cells infiltration, and clinical outcome was achieved through TIMER analysis. There was no significant correlation observed between *CCNB1* and 6 kinds of immune cells infiltration (Fig. 6j). Furthermore, no significant correlation was observed between infiltration status and cumulative survival (Fig. 6k).

## Discussion

In the present study, we achieved gene microarray data from GEO database and identified DGEs between ESCA patients and normal people. WGCNA was performed to reveal key modules with clinical significance, and greenyellow module was screened out through preservation evaluation. Meanwhile, we applied GO and KEGG analysis to research the mainly correlated biological pathway of the hub module. After that, intersection genes were extracted between greenyellow module and DEGs, and a PPI network was built. Finally, *CCNB1* was selected and was verified expression and prognostic value through multiple tests.

GO analysis indicated that the genes in greenyellow module primarily participate in such pathways, including chromosome segregation, mitotic nuclear division, chromosomal region, helicase activity, and structural constituent of cytoskeleton. Some of the pathways have been proved in former studies [21–23]. KEGG pathway analysis found that oocyte meiosis, cellular senescence, progesterone-mediated oocyte maturation, and cell cycle were markedly enriched, and recently Shao et al. also reported important enriched pathways of cellular senescence in ESCC [24, 25].

It is well-known that *CCNB1* is involved in cell proliferation by binding to *CDK1* to form a complex [26]. The target substrates were phosphorylated by the *CCNB1*-*CDK1* complex, which leads to cell cycle progression. Many important steps in mitosis will be initiated by the activated *CCNB1*-*CDK1* complex, including condensation of chromosomes, assembly of spindle pole, and breakdown of nuclear envelope [27, 28], which is consistent with our results. The enrichment analysis suggests that the key module, including *CCNB1* in the WGCNA, plays a vital role in mitosis. Furthermore, abnormal *CCNB1* expression is often associated with a variety of cancers [29–31], and *CCNB1* can be used as a biomarker to predict cancer. However, there have been few reports on the direct correlation between *CCNB1* and esophageal cancer, and our research has made important explorations in this area.

Jiang et al. reported that oridonin could downregulate the *CCNB1* to arrest the cell cycle in ESCA [32]. Zhang et al. reported that secalonic acid D induced cell G2/M phase arrest by downregulating the expression of *CCNB1* and increasing the phosphorylation of *CDK1* [33]. Zeng et al. [34] also reported a similar conclusion that *EPB41L3* was a potential ESCA suppressor gene and induced G2/M cell cycle arrest by activating *CDK1*/*CCNB1* signaling. Therefore, these studies all suggested that *CCNB1* was correlated with ESCA and clarified the feasibility of a biomarker in ESCA. However, the current studies all focused on mediating the upstream gene of *CCNB1* to inhibit the ESCA. Our study focused on the analysis of the role of *CCNB1* as a biomarker on ESCA expression and prognosis and achieved good results. In addition, many studies also reported that the sensitivity to chemoradiotherapy for ESCA would increase via arresting the cell cycle at the G2/M phase and downregulating expression levels of *CCNB1* and *CDK1 *[35–37].

In conclusion, based on DEGs and key modules related to esophageal cancer, *CCNB1* was identified as the hub gene, which offered novel insights into the development and treatment of esophageal cancer.

## Supplementary Information

**Additional file 1: Figure S1.** Volcano plot of up-regulated and down-regulated DEGs in GSE20347(A), GSE29001(B), and GSE111044(C).

**Additional file 2: Figure S2.** Venn diagram. Venn diagram indicated overlapping 39 hub genes of the DEG and WGCNA.

## Data Availability

The datasets used and/or analyzed during the present study are available from the corresponding author on reasonable request.

## Abbreviations

DEGs: Differential expression genes
WGCNA: Weighted gene co-expression network analysis
ESCA: Esophageal cancer
ESCC: Esophageal squamous cell carcinoma
CCNB1: Cyclin B1
MM: Module membership
GS: Gene significance
PCA: Principal component analysis
GO: Gene Ontology
BP: Biological process
CC: Cellular component
MF: Molecular function
KEGG: Kyoto encyclopedia of genes and genomes
PPI: Protein–protein interaction
HCMDB: Human cancer metastasis database
KM: Kaplan–Meier
