# Supplementary figures and images for: Systematic identification of key functional modules and genes in esophageal cancer
Source: Cancer Cell Int. 2021 Feb 25;21:134. doi: 10.1186/s12935-021-01826-x (PMC7905886; doi:10.1186/s12935-021-01826-x)

A

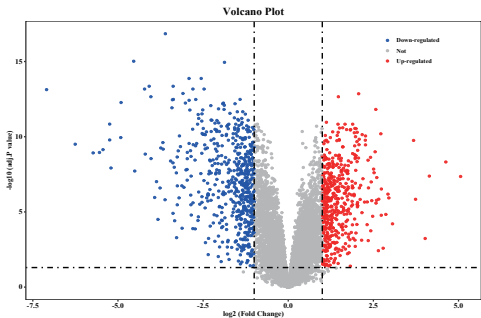

B

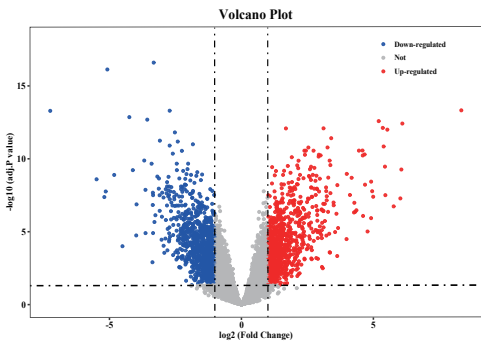

C

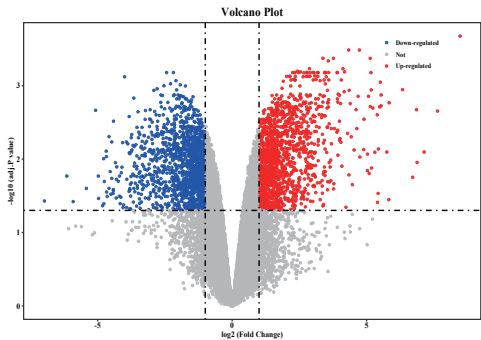

Supplement: Supplementary file 1 — Additional file 1: Figure S1. Volcano plot of up-regulated and down-regulated DEGs in GSE20347(A), GSE29001(B), and GSE111044(C). [file 12935_2021_1826_MOESM1_ESM.pdf]

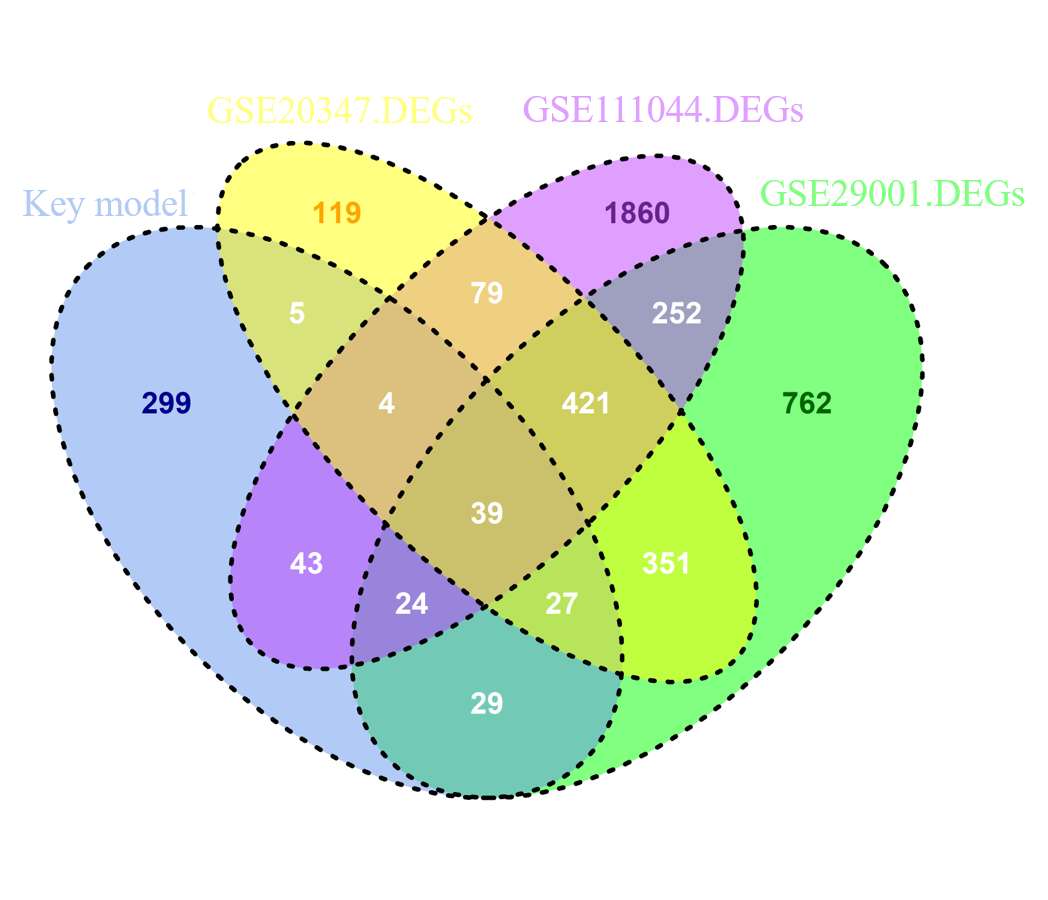

Supplement: Supplementary file 2 — Additional file 2: Figure S2. Venn diagram. Venn diagram indicated overlapping 39 hub genes of the DEG and WGCNA. [file 12935_2021_1826_MOESM2_ESM.tif]
